# Rehabilitation healthcare professionals’ competence and confidence in differentially diagnosing deafblindness from autism spectrum disorders: a cross-sectional survey in South Africa

**DOI:** 10.1186/s12909-022-03258-1

**Published:** 2022-03-21

**Authors:** Nomfundo Moroe, Khetsiwe Masuku, Lebogang Shirame

**Affiliations:** Department of Speech Pathology and Audiology, University of the Witwatersrand, Johannesburg, South Africa

**Keywords:** Autism spectrum disorder, Competence, Deafblindness, Differential diagnosis, Rehabilitation

## Abstract

**Background:**

Early diagnosis and management of children who are deafblind is important to alleviate the effects of deafblindness on the development of the child who is deafblind and their families. However, children who are deafblind are often misdiagnosed or diagnosed late. The misdiagnosis or late diagnosis has been attributed to many factors, one of which is the competence and confidence of healthcare professionals in differentially diagnosing deafblindness from other conditions, in most cases, autism spectrum disorder (ASD). The study therefore aimed to establish the competence and confidence of rehabilitation healthcare professionals in differentially diagnosing deafblindness from ASD in the South African context.

**Methods:**

A cross-sectional survey design was employed for the study. An online questionnaire was distributed to rehabilitation healthcare professionals (*N* = 78) via Survey Monkey. Data were analyzed using descriptive and inferential statistics. Ethical clearance and permission were obtained from relevant stakeholders prior to the commencement of the study.

**Results:**

Regarding the rehabilitation healthcare professionals in this study, 54% were competent in diagnosing ASD, while only 35% could correctly diagnose deafblindness. In some instances, symptoms were classified as associated with both ASD and deafblindness, when they were just those of deafblindness. Of all the rehabilitation healthcare professionals in this study, speech language therapists displayed the most knowledge of deafblindness. Furthermore, healthcare professionals who had between one and nine years of working experience had more knowledge of deafblindness than other professionals with more or less experience.

**Conclusion:**

Deafblindness is often underdiagnosed or misdiagnosed as ASD. This is due to the lack of competence and confidence of rehabilitation healthcare professionals in diagnosing it. The findings therefore highlight the need for training of rehabilitation healthcare professionals. Training on deafblindness could be included as part of the curriculum in the various undergraduate programs. Deafblindness could also form part of the Continuous Professional Development (CPD) training programs at various healthcare facilities. A team approach to the training would be ideal as it would facilitate peer learning and support. More research is required as it would inform evidence-based assessment, and management and support strategies for children who are deafblind and their families.

## Background

Deafblindness is a distinct disability resulting from a dual sensory (hearing and vision) impairment of a severity that hinders the senses from compensating for each other. This disability limits a person’s activities and greatly restricts full participation in society. Therefore, society is required to compensate by means of specific services, environmental alterations and/or technology [1, 2]. Deafblindness is a lifelong dual sensory impairment and it ranges from mild to total deafness and blindness, depending on the various combinations [3]. It significantly affects communication, socialization, orientation and mobility, access to information and daily living [3]. Deafblindness can be congenital or acquired. The current study is concerned with congenital deafblindness [4–7]. Congenital deafblindness is a rare neurodevelopmental disorder. Neurodevelopmental disorders are said to be the leading cause of disability globally. They have a profound impact on the quality and duration of life [8]. Neurodevelopmental disorders may manifest through functional limitations in cognition, vision, hearing and neuromotor ability [8], features that are associated with deafblindness. Congenital deafblindness is caused by a number of factors, including CHARGE syndrome, prematurity, meningitis, cytomegalovirus and rubella (in countries with no routine immunization programmes) as the most common causes [7].

Globally, the prevalence of deafblindness is low and it is currently estimated at 0.2 to 2% [2, 6]. Specifically in South Africa, which is regarded as an upper middle income country, the prevalence of deafblindness is estimated at 0.1in the 5–17 age group [2]. Despite deafblindness not being recognized as a distinct disability in most low and middle income countries (LMIC), the prevalence of diagnoses may increase due to advancement in medical services [9].

Deafblindness is often misdiagnosed or diagnosed late [10]. Literature attributes the misdiagnosis and/or late diagnosis of congenital deafblindness to several factors, including: i) the low prevalence of congenital deafblindness as mentioned above. ii) the heterogeneity of the population due to different degrees of vision and hearing, different modes of communication and comorbidities and causes of deafblindness [6]. iii) the combined sensory loss, which results in difficulties in using traditional functional assessments and psychological tests which often require full sensory functioning [6]. iv) the communication system used, which often results in communication difficulties during assessment. v) the interpretation of deafblind behaviour, often called blindism [6]. vi) the narrow or discipline focus in the management instead of a team approach and vii) the lack of a consensus on the definition owing to two definitions – a medical and a functional definition. The medical definition is concerned with audiological and visual criteria, while the functional definition is concerned with self-reports and observations, evaluating the individual impact of vision and hearing loss on everyday life activities and the individual’s possibilities of participation [4, 6]. This lack of consensus contributes to the insufficient understanding of deafblindness as a condition amongst healthcare professionals [11]; viii) other disabilities tend to mask deafblindness [12] and its close resemblance to autism spectrum disorder (ASD) [13].

Deafblindness is often misdiagnosed as ASD [7, 13–15] because fundamentally, both conditions affect the way sensory information is accessed and processed [13, 14]. Deafblindness is a dual sensory (hearing and vision) loss, which manifests through auditory and visual processing problems. On the other hand, ASD manifests through difficulties in processing auditory and visual stimulation due to the way the brain processes sensory information, rather than sensory loss [13]. Ultimately, both conditions affect access to information [14], communication, social interaction and behavior (children tend to present with restricted and repetitive behaviors) [7, 13]. Both of these conditions also significantly affect vocational and future education achievements [7]. Furthermore, the concurrent visual and hearing impairment limits access to information required for the development of language, communication, cognition, socio-emotional and mobility skills and abilities [3, 16, 17]. The absence of the abovementioned skills negatively impacts social participation and educational outcomes [16, 18]. To facilitate the development of these skills and to mitigate the impact of deafblindness on the developing child and their family, early identification and intervention, especially in the first year of life, are essential [19].

Early intervention refers to the timely identification and management of children between birth and three years of age who are at risk or have an established risk for a developmental delay [20]. A misdiagnosis or late diagnosis not only negatively impacts on the development of the child, but it deprives their families of much-needed resources and timely support [21]. Early identification and intervention i) directly determine whether the underlying medical impairments will progress into a disabling condition or help reduce the risks that children who are deafblind would otherwise encounter; ii) facilitate long-term benefits for families and societies by minimizing mental distress of families and the risk of the child requiring more intensive care in the future; and iii) promote parent–child bonding and enhance the caregiving process for the child [9].

Paul [9] laments the lack of availability of trained professionals in early intervention services for children who are deafblind. This directly affects their early diagnosis and management. Rehabilitation healthcare professionals, specifically audiologists, speech and language therapists, occupational therapists and physiotherapists, make up the healthcare team of professionals trained to provide early identification and intervention to children who are deafblind [22]. Holte [23] posits that rehabilitation health professionals need to possess adequate knowledge and skills to identify and diagnose deafblindness and to recognize risk factors that potentially predispose children to deafblindness from their medical and family history. The services needed to cater for the unique needs of each child include family counselling, needs based training support by trained educators, provision of necessary aids and appliances and continued medical support, including audiology, family training, physiotherapy, occupational therapy, clinical psychologist services, nutrition services, counselling, home visits, assistive technology, speech language therapy, and special education training [9].

As alluded to earlier, literature has shown that deafblindness may be misdiagnosed as ASD. To this end, Belote [13] developed a checklist of key ASD features which are often confused with deafblindness. These include delays in verbal and non-verbal communication; delays in developing social interaction skills; restricted areas of interest; use of repetitive speech or engaging in repetitive activities or routines; stereotyped movements or behaviors; resistance to environmental change; resistance to change in daily routines; unusual responses to sensory experiences and difficulties with executive function skills. Understanding that deafblindness and ASD are closely related, it is important to establish the rehabilitation healthcare professional’s competence in differentially diagnosing deafblindness from ASD. In the South African context, there is no documented evidence on the competence and confidence of rehabilitation health professionals in differentially diagnosing deafblindness and autism.

Buys [24] argues that conceptually, it is not easy to define professional competence. This is because professional competence is the combination of the person's knowledge, skills and clinical judgment, experience and attitude required to face occupational or environmental pressures and demands [25, 26]. For the purposes of this study, professional competence refers to the person’s knowledge, skills and clinical judgment over the years (experience).

## Research aim

The main aim of this study was to establish rehabilitation healthcare professionals’ competence and confidence in differentially diagnosing deafblindness from ASD in the South African context.

## Objectives

- To establish whether participants can match the provided symptoms with the described condition for a differential diagnosis – deafblindness vs ASD
- To evaluate the effect of years of experience on the correct differential diagnosis of deafblindness
- To compare the levels of perceived knowledge according to the professional groups – (occupational therapists (OTs); audiologists (AUDs); speech therapists (STs), physiotherapists (PTs) and speech therapists and audiologists (STA)).

## Methods

### Research design

This study employed a cross-sectional survey design [27] to allow the researchers to obtain information from a cohort of rehabilitation healthcare professionals at a given point in time. Adhered to the World Medical Association (WMA) Declaration of Helsinki (2013) ethical guidelines, participants were furnished with the information letter highlighting the study’s details and requirements. Furthermore, informed consent was obtained from participants prior to participating in the study. Participants were made aware that participation is voluntary, and they can withdraw from the study at any point without any penalties. Lastly, participants were informed that there is no risk or direct benefits in participating in this study. Ethics clearance was obtained from the University’s Human Research Ethics Committee (HREC) (non-medical) (Protocol Number: STA_2020_21). Potential participants were recruited from the various rehabilitation health- care professional boards, including South African Speech Language and Hearing Association (SASLHA), South African Audiology Association (SAAA), Occupational Therapy Association of South Africa (OTASA) and Physiotherapy Association of South Africa (PASA). Once permission to distribute the online questionnaire via the respective portals was granted, the link to the study was forwarded to the representatives of the above-mentioned boards to send to potential respondents.

### Data collection method

Data were collected via a self-administered online questionnaire. The questionnaire was developed from the checklist developed by Belote [13] on the key features of ASD which are often confused with deafblindness. The questionnaire described behaviors associated with each feature, and respondents were required to state if the feature described deafblindness or ASD. The responses to the questions included the following options: “deafblindness”; “autisms”; “both”; and “I do not know”.

Prior to the commencement of the study, the questionnaire was sent to two AUDs, two OTs, two STAs and three PTs to comment on the content and clarity of the questionnaire, and to provide appropriate suggestions for improvements [27]. The questionnaire was emailed together with a suggestion form. The final version of the questionnaire was uploaded onto the Survey Monkey and forwarded to the professional boards for further distribution to potential respondents. The study link was active for six weeks, from 18 August to 30 September 2020.

### Participants

Non-probability purposive sampling was used to recruit and select participants [27]. Inclusion criteria included rehabilitation healthcare professionals registered with the Health Professions Council of South Africa (HPCSA) and with a working experience of six months and more. In total, 78 respondents participated in this study (Table 1).

**Table 1:** Descriptive characteristics of the study participants

Variable	Category	Frequency	Percentage
Gender	Female	66	84.62
	Male	8	10.26
	Undisclosed	4	5,12
Profession	Audiologist	16	20.51
	Occupational therapist	19	24.36
	Physiotherapist	5	6.41
	Speech therapist	12	15.38
	Speech therapist and audiologist	24	30.77
	Researchers	2	2.56
Institutions	UKZN	12	15.38
	University of Cape Town	18	23.08
	University of Pretoria	10	12.82
	University of Witwatersrand	22	28.21
	Northwest University	16	20.51
Working with pediatric or adults population	Adult	10	12.82
	Pediatric	19	24.36
	Both	44	56.41
	None (2 Researchers/ 3 did not disclose)	5	6.41
Working	Public hospital		
	Yes	30	38.46
	No	48	61.54
	Private hospital		
	Yes	20	25.64
	No	58	74.36
	Private practice		
	Yes	6	7.69
	No	72	92.31
	School		
	Yes	14	17.95
	No	64	82.05
	Institution of higher learning		
	Yes	15	19.23
	No	63	80.77
	Other		
	Yes	10	12.82
	No	68	87.18
Province	Eastern Cape	3	3.85
	Gauteng	38	48.72
	Kwa-Zulu Natal	11	14.10
	Limpopo	3	3.85
	Mpumalanga	3	3.85
	Northwest	7	8.97
	Northern Cape	3	3.85
	Western Cape	10	12.82

### Data analysis

Data were collected via Survey Monkey and were downloaded and imported to an Excel spreadsheet for analysis. Descriptive statistics were used to analyze the data [28]. Specifically, frequency tables and graphs were used to summarize the data. Inferential statistics were used to determine if there was any association between variables of interest such as knowledge levels and participant’s profession, with p-value set at 0.05. A proportional test was also used to determine if there was any association between rehabilitative healthcare professionals’ performance and years of experience.

## Results

### Descriptive results

**Fig. 1:**
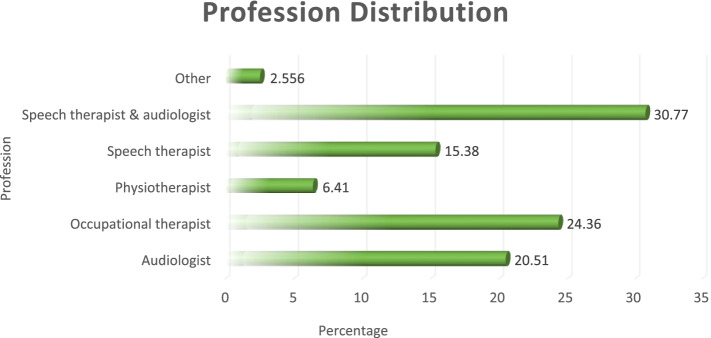
Participants’ profession distribution

A total of 78 participants responded and were enrolled in this study. All the participants were included in the study as they met the inclusion criteria. However, it was observed that some participants skipped some of the questions pertaining to differential diagnoses. Since the only available options were: “deafblindness”; “autism”; “both”; and “I do not know”, all the skipped questions were included in the “I do not know” option, which seemed to be the most suitable one to account for the skipped questions. The descriptive characteristics of the participants are summarized in Table 1. There were more females (*n* = 66, 89.19%) than male participants (Table 1). Most of the participants were speech therapists and audiologists (*n* = 24, 30.77%) with physiotherapists being the least represented (*n* = 5, 6.41%) while others were audiologists (*n* = 16, 20.51%) and *n* = 2, 2.56% researchers (Fig. 1).

**Fig. 2:**
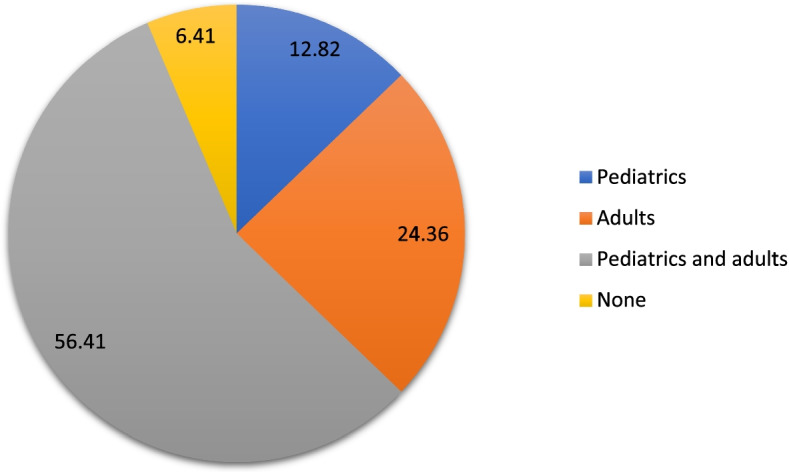
Distribution of caseload population

Most participants acquired their qualification from the University of the Witwatersrand (*n* = 22, 28.21%). There were 56.41% (*n* = 44) participants who were working with both pediatric and adult populations (Fig. 2). None referred to the reseachers (2.56%) and the rest (3,85%)did not discolse.

**Fig. 3:**
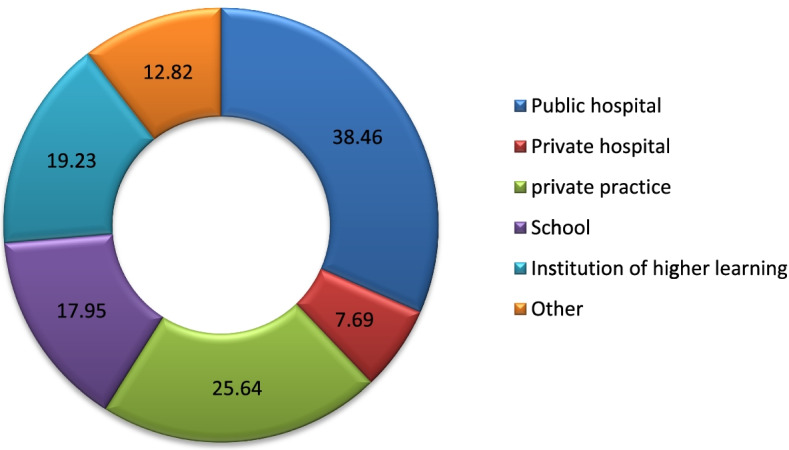
Work distributions of the participants

Most of the participants were working in public hospitals (*n* = 30, 38.5%), while 25.64% (*n* = 20) were working in private institutions (Fig. 3). Other referred to those who are unemployed (*n* = 1) and retired (*n* = 1).Most of the participants were working in the Gauteng province (*n* = 38, 48.72%).

**Fig. 4:**
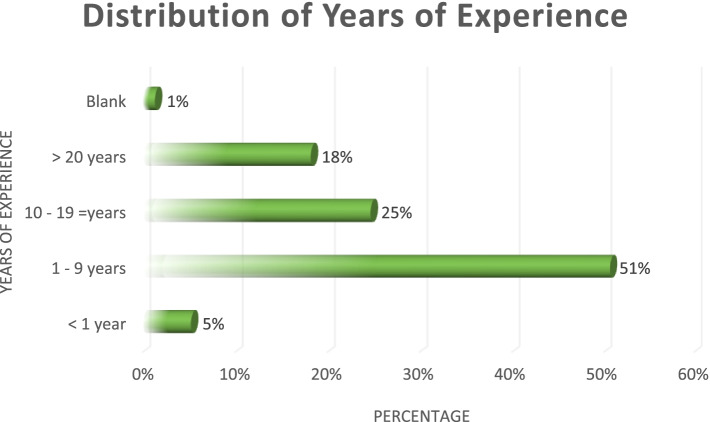
Distribution of years of experience

The median practicing time was nine years, with an interquartile range of 3.75–15.5 years. The median experience time was eight years, with an interquartile range of 4–15 years (Fig. 4). O

### Autism and deafblindness knowledge responses

#### Autism

**Fig. 5:**
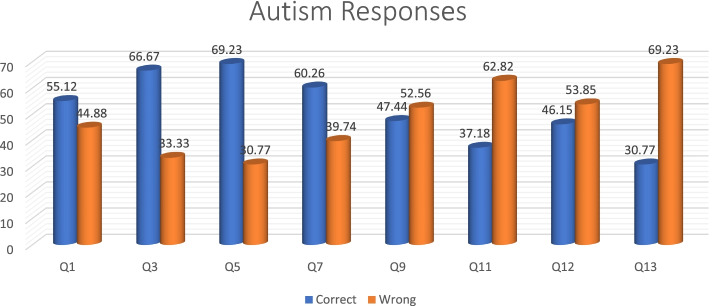
Distribution of Autism responses

Knowledge about autism condition, based on the questions asked, is summarized in Table 2. There were eight different questions where the correct answer was autism condition. More than 50% of the participants got Q1 (55.12%), Q3 (66.67%), Q5 (69.23%) and Q7 (60.26%) answers correct, while Q9 (47.44%), Q11 (37.44%), Q12 (46.15%) and Q13 (30.77%) were missed by more than 50% (Fig. 5). A significant proportion of participants, ranging from 24.36% to37.18%, indicated that they did not know the correct response for all the questions.

**Table 2:** Knowledge questions specifically for autism

Question	Autism (correct)	Deafblindness	Both	I don’t know
	**n (%)**	**n(%)**	**n (%)**	**n (%)**
Q1	43 (55.12)	5(6.41)	10(12.82)	20(25.64)
Q3	52(66.67)	3(3.85)	4(5.13)	19(24.36)
Q5	54(69.23)	3(3.85)	0	21(26.92)
Q7	47(60.26)	5(6.41)	6(7.69)	20(25.64)
Q9	37(47.44)	5(6.41)	16(20.51)	20(25.64)
Q11	29(37.18)	5(6.41)	21(37.18)	23(29.49)
Q12	36(46.15)	6(7.69)	14(17.95)	22(28.21)
Q13	24(30.77)	6(7.69)	19(24.36)	29(37.18)

**Fig. 6:**
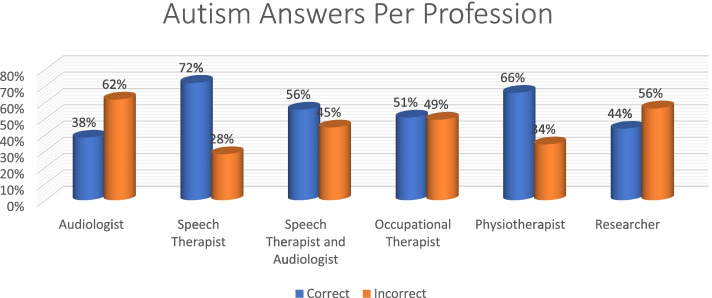
Distribution of Autism responses per profession

Speech therapist had the most correct answers (72%), with audiologists scoring the lowest in the diagnosis of Autism (Fig. 6).

**Fig. 7:**
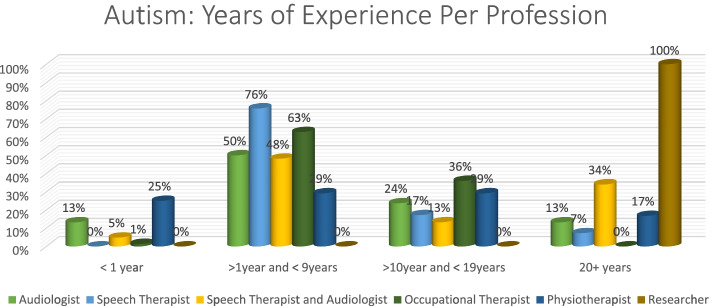
Distribution of Years of experience per profession

In terms of years of experience, those with more than one year and less than nine years’ experience, had the most correct answers (76%), while researchers scored 100% in the 20 + year group (Fig. 7).

#### Deafblindness

**Fig. 8:**
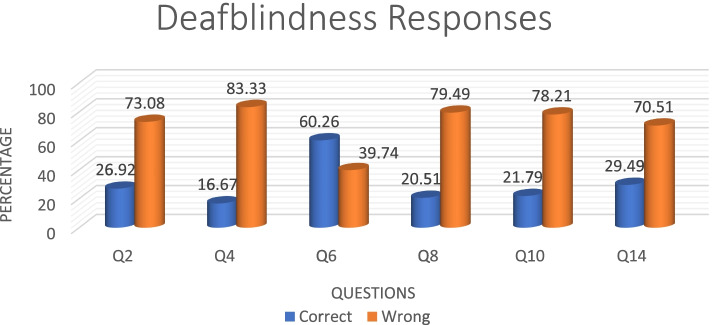
Distribution of deafblindness responses to assess deafblindness competence

Knowledge about the condition of deafblindness, based on the questions asked, is summarized in Table 3. There were six different questions, where the correct answer was deafblindness condition. More than 50% of the participants got Q6 (60.26%) answers correct, while Q2 (26.92%), Q4 (16.67%), Q8 (20.51%), Q10 (21.79%) and Q14 (29.49%) were missed by more than 50% (Fig. 8). A significant proportion of participants, ranging between 29.49% and 33.33%, indicated that they did not know the correct response for all the questions.

**Table 3:** Knowledge questions specifically for deafblindness

Question	Deafblindness (correct)	Autism	Both	I don’t know
	**n(%)**	**n(%)**	**n(%)**	**n(%)**
Q2	21 (26.92)	13(16.67)	21(26.92)	23(29.49)
Q4	13(16.67)	31(39.74)	9(11.54)	25(32.05)
Q6	47(60.26)	4(5.13)	4(5.13)	23(29.49)
Q8	16(20.51)	16(20.51)	15(19.23)	31(30.74)
Q10	17(21.79)	16(20.51)	20(25.64)	25(32.05)
Q14	23(29.49)	7(8.97)	22(28.21)	26(33.33)

**Fig. 9:**
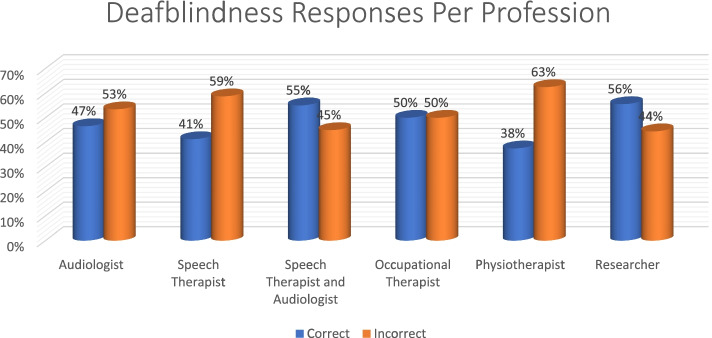
Distribution of deafblindness responses per profession

Professionally, speech therapists (59%) had the most correct answers for deafblindness, while physiotherapists had the most incorrect answers (38%). Occupational therapists had an equal split (Fig. 9).

**Fig. 10:**
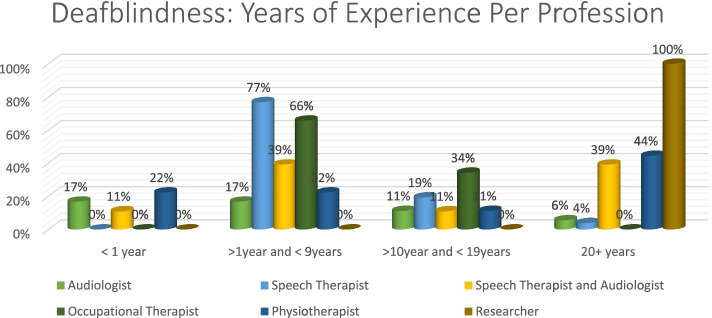
Distribution of years of experience per profession

Once again, speech therapists (77%) had the most correct answers in the > 1 year and < 9 years group. All professions performed poorly in the < 1 year category, with physiotherapists obtaining 22% in that category. In the 20 + category, researcher obtained 100% (Fig. 10).

**Fig. 11:**
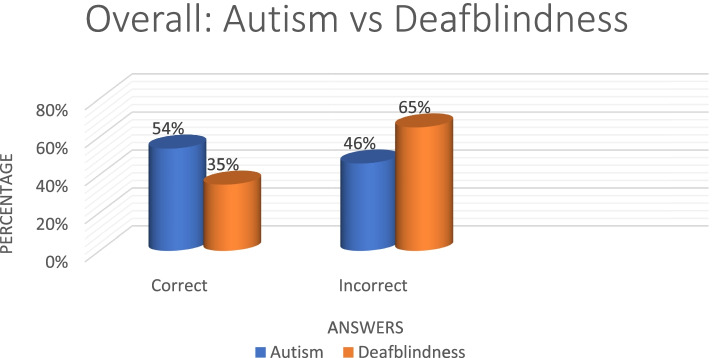
Autism vs deafblindnes

**Fig. 12:**
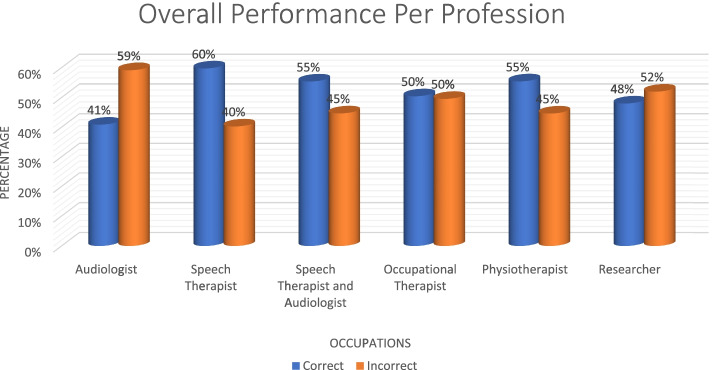
Distribution of overall performance per profession

**Fig. 13:**
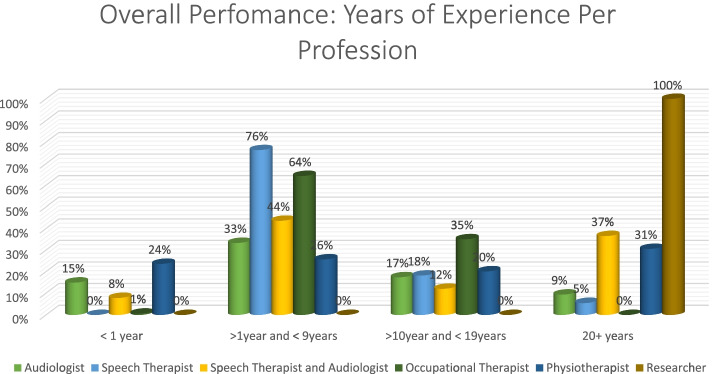
Distribution of overall performance: years of experience per profession

Overall, autism had the most correct responses at 54% while deafblindness was at 35% (Fig. 11). Speech therapists had the most correct answers (60%) with audiologists performing poorly at 41% (Fig. 12). The 20 + had the most correct (*n* = 2, 100%) as obtained by researchers. The > 1 year and < 9 years group was the second highest with the < 1 performing the poorest (Fig. 13).

### Knowledge scores for autism

The median autism score was 5, with an interquartile range of 1–6 scores. There was no significant correlation between autism score and years of experience as defined by a positive weak correlation coefficient of 0.0467 (*p*-value = 0.9769) or number of years in practice, as defined by the negative weak correlation coefficient of 0.0034 (*p*-value = 0.6886). The median autism score was not significantly different between males and females (*p*-value = 0.3406). Comparing the autism median scores by profession, there was no significant difference in the median score (*p*-value = 0.4146).

**Fig. 14:**
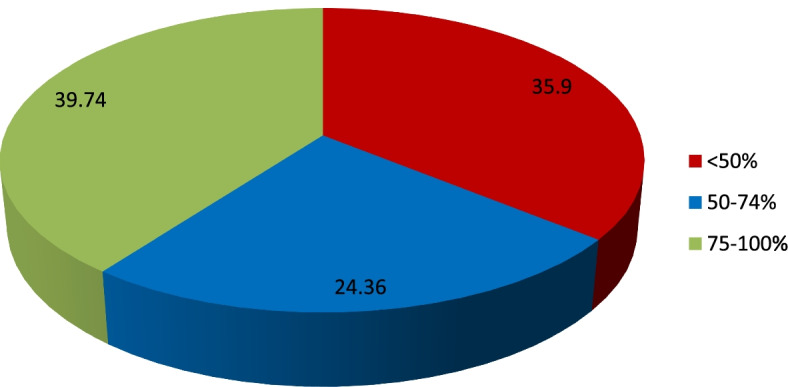
The autism score distribution: poor (< 50%), moderate (50–74%) and good/excellent (75–100%)

Further categorizing the autism score in three groups, there were 35.9% (*n* = 28) participants with poor knowledge for autism, 24.36% (*n* = 19) with moderate/average knowledge and 39.74% (*n* = 31) with good/excellent knowledge for autism condition (Fig. 14). There was no association between autism knowledge levels and gender (*p*-value = 0.657). Similarly, there was no association between autism knowledge levels and participants’ professions (*p*-value = 0.473). The median years of practice (*p*-value = 0.4492) and the median number of years of experience (*p*-value = 0.8971) were not significantly different between the three autism knowledge levels.

### Knowledge scores for deafblindness

**Fig. 15:**
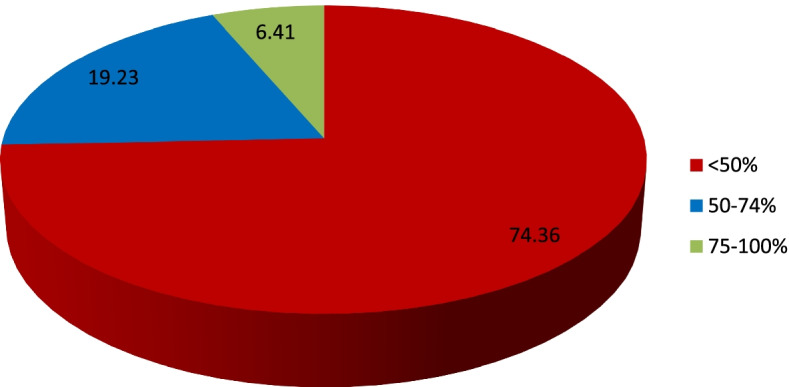
The deafblindness score distribution poor (< 50%), moderate (50–74%) and good/excellent (75–100%)

The median deafblindness score was 1 with an interquartile range of 0–3 scores. There was no significant correlation between deafblindness score and years of experience as defined by a positive weak correlation coefficient of 0.0855 (*p*-value = 0.4598) or number of years in practice, as defined by the positive weak correlation coefficient of 0.0903 (*p*-value = 0.4379. The median deafblindness score was not significantly different between males and females (*p*-value = 0.95). Comparing the deafblindness median scores by profession, there was no significant difference in the median score (*p*-value = 0.6569. There were 74.36% (*n* = 58) participants with poor knowledge for deafblindness, 19.23% (*n* = 15) with moderate/average knowledge and 6.41% (*n* = 5) with good/excellent knowledge for deafblindness condition (Fig. 15).

There was no association between deafblindness knowledge levels and gender (*p*-value = 0.676). Similarly, there was no association between deafblindness knowledge levels and participants’ professions (*p*-value = 0.492). The median years of practice (*p*-value = 0.6336) and the median number of years of experience (*p*-value = 0.07143) were not significantly different between the three deafblindness knowledge levels.

### Knowledge scores for autism and deafblindness

There was a significant difference between the median knowledge scores for autism and deafblindness in this study (*p*-value < 0.001). The participants showed more knowledge of autism than deafblindness. However, in general, there was a direct correlation in the autism and deafblindness score with a moderate positive correlation coefficient of 0.4745 which was overwhelmingly significant (*p*-value < 0.001). This means that as the knowledge levels of autism increase, the knowledge score for deafblindness also increases.

The overall median score for combined deafblindness and autism assessment was 7 with an interquartile range of 2–8 scores. There was no significant correlation between combined deafblindness and autism score and years of experience as defined by a positive weak correlation coefficient of 0.0719 (*p*-value = 0.5346) or number of years in practice, as defined by the positive weak correlation coefficient of 0.0994 (p-value = 0.3929. The median combined deafblindness and autism score was not significantly different between males and females (*p*-value = 0.5453). Comparing the combined deafblindness and autism median scores by profession, there was no significant difference in the median score (*p*-value = 0.4831).

**Fig. 16:**
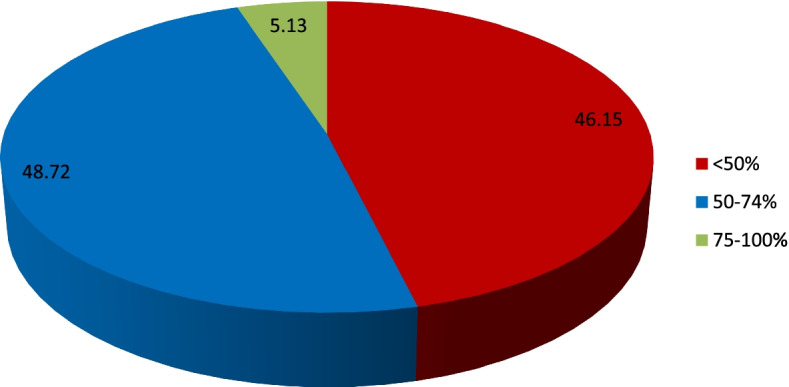
The combined autism and deafblindness score distribution poor (< 50%), moderate (50–74%) and good/excellent (75–100%)

There were 46.15% (*n* = 36) participants with poor knowledge for both autism and deafblindness, 48.72% (*n* = 38) with moderate/average knowledge and 5.13% (*n* = 4) with good/excellent knowledge for deafblindness condition (Fig. 16). There was no association between deafblindness knowledge levels and gender (*p*-value = 0.774). Similarly, there was no association between the combined deafblindness and autism knowledge levels and participants’ professions (*p*-value = 0.414). The median years of practice (*p*-value = 0.6755) and the median number of years of experience (*p*-value = 0.8085) were not significantly different between the combined deafblindness and autism knowledge levels.

## Discussion

This study sought to determine the perceived competence and confidence of rehabilitation healthcare professionals (audiologists, speech language therapists, speech language therapists and audiologists, physiotherapists and occupational therapists) in differentially diagnosing children who are deafblind, in the South African context.

The early differential diagnosis of deafblindness is imperative to ensure that specialized interventions are implemented as soon as possible to mitigate the impact of deafblindness on the different areas of child development [29, 30]. Due to the different areas of development that are impacted by the diagnosis of deafblindness, an integration of clinical expertise approaches from early diagnosis to management of deafblindness becomes key [18, 30].

To be able to competently diagnose and manage children who are deafblind, it is important that the early intervention team possess the necessary competence and skills to differentially diagnose deafblindness from other neurodevelopmental disorders as these may potentially mask deafblindness [12].

Current findings suggest that approximately 54% of the rehabilitation healthcare professionals surveyed were competent in diagnosing ASD, while only 35% of them could correctly diagnose deafblindness. Hoevenaars-van den Boom [14] argues that autism is over- diagnosed in individuals with sensory impairment due to the topographical similarities in behaviors but differences in the underlying mechanisms or processes that cause those behaviours. Deafblindness is a multisensory disability. Therefore, it is possible that participants in this study over-diagnosed autism over deafblindness due to the similarities mentioned above. Our findings are further supported by Davidovitch [31], who posits that ASD is over- diagnosed in relation to other neurodevelopmental disorders.

In relation to deafblindness, there is an increase in autism awareness and a plethora of research on ASD in South Africa, because of advocacy for services for children with autism. On the other hand, there is limited research, training, local literature, and services and material in the area of deafblindness [7]. Therefore, it is possible that participants in this study had a better understanding of ASD.

Findings of the study also indicated that of all the rehabilitation healthcare professionals who participated in the study, only speech therapists (60%) possessed the necessary competence and confidence to diagnose deafblindness. Dammeyer & Larsen [32] and Parker [33] submit that communication is one of the significant challenges for individuals who are deafblind, often negatively affecting their participation in different aspects of life. It is therefore expected that they would know how to diagnose and manage deafblindness. The poor competence and confidence demonstrated by the other rehabilitation healthcare professionals in the study suggest that the curriculum of other rehabilitation healthcare professionals in South Africa may not necessarily be providing sufficient and specific content for honing rehabilitation healthcare professionals’ knowledge, skills and confidence, and their ability to provide early diagnosis and management services to children who are deafblind and their families. Training may be from a general perspective and not necessarily condition specific, yet the uniqueness of deafblindness requires professionals to possess condition specific knowledge and skills. Moroe [34]; Paul et al.[9]; Jaiswal et al. [3]; Wittich et al. [22], submit that health professionals in LMIC are not adequately trained to detect and provide intervention services to children with multisensory impairment, including deafblindness, often compromising their care.

Healthcare professionals who had between one and nine years of working experience had more knowledge on deafblindness than other professionals who had worked for less than a year or 10 years and more. These findings are consistent with the results of a study conducted by Sasaki [35] on competencies of nurses, where nurses with < 5 years of clinical experience had significantly more confidence than the nurses with only one year of experience. Arguably, the cohort with more than 10 years of experience performed poorly compared to the cohort that had one to nine years of experience, due to a dearth of knowledge on deafblindness locally [10, 34], and the even lower prevalence of deafblindness a decade ago. Interestingly, Christen [36] argues that although experience is necessary, it is not synonymous with being an expert. This was also observed by Benner [37], who noted that the number of years on the job may develop competence; however, the passage of time and occurrence of events does not automatically esult in an expert status. Bobay [38] purports that those years of experience, while they may facilitate fluidity and flexibility, do not necessarily develop complex reflexive thinking, which is considered an important component of clinical expertise. In this study, the current authors attribute the decline in competence with more years of experience to the lack of knowledge previously, compared to the increase in knowledge, albeit slight, in recent years. Another consideration may be anecdotal evidence of improved curriculum in recent years.

### Recommendations

Children who are deafblind require a variety of therapeutic services to facilitate functioning and participation. A multi- or interdisciplinary rehabilitation approach to the management of children who are deafblind is needed if appropriate services are to be provided to them. Comprehensive rehabilitation service delivery is a crucial aspect of the continuum of care, yet it is often hindered by, amongst many factors, the lack of competence and confidence of rehabilitation healthcare professionals in some areas [39]. In this case, the area is deafblindness. We therefore call for existing institutions that train rehabilitation healthcare professionals to include deafblindness as a course in their curriculum so that healthcare professionals can become competent in assessing and managing this population. Course content should include the causes of deafblindness, communication requirements, and functioning and participation [2]. Training could also be done as part of multi-disciplinary meetings at healthcare facilities.

Rehabilitation healthcare professionals who are already in the field need to capacitate themselves in knowledge on deafblindness to improve their competence and confidence in assessing and managing children who are deafblind. They therefore need to attend continued professional development courses on deafblindness. Institutions of higher education that train rehabilitation healthcare professionals need to be deliberate in providing training opportunities by providing short courses and continued professional development courses on deafblindness.

There is a clear dearth of research around deafblindness in South Africa. The lack of research contributes to lack of knowledge in the area, which in turn influences the management of deafblindness. This study advocates for the building of research capacity around deafblindness to facilitate the availability of robust context specific, but globally relevant, evidence on rehabilitation in the field of deafblindness in South Africa. Multi-disciplinary research is specifically proposed because it would bring about different and important perspectives.

Paul et al. [9] state that providing sustainable integrated early intervention services for children who are deafblind requires substantial commitment of effort, time and resources. It is therefore recommended that rehabilitation healthcare professionals invest time and effort in upskilling themselves to be able to identify and diagnose children at risk of deafblindness at an early age. In that way, they could provide them with the necessary early intervention services on time. and develop multi-disciplinary family centered rehabilitation programs that would address all the needs of the person who is deafblind and their families. Multi-disciplinary family centered rehabilitation programs need to be implemented at all three levels of care, namely, primary, secondary and tertiary care. Furthermore, Paul et al. [9] insist that early identification screening should not be condition specific and should shift from the narrow discipline focus to a team approach. This can be achieved by screening children for any at risk symptoms, while recognizing deafblindness as a separate disability. This would increase opportunities for children who are deafblind to receive early identification and intervention and reasonable accommodation [9]. Thus, there is a need to develop tests capable of differentiating deafblindness from ASD [14] and the involvement of all the applicable rehabilitation professionals.

### Limitations

The different professional bodies were the gatekeepers in that they sent the survey link to their members without the involvement of the researchers. Furthermore, professional members who are not affiliated with these bodies, were potentially excluded from the study. It is also possible that some professionals may have had limited or no access to the online platforms. Hence, they did not participate. Lastly, the study was quantitative; therefore, we cannot comment qualitatively on the reasons why some participants performed poorly in this task. There is a need to follow up to understand the factors contributing to the participants’ competence and confidence in differentially diagnosing deafblindness from ASD.

### Strengths of the study

This study included various rehabilitation healthcare professionals who are ideally the multidisciplinary team responsible for identifying and managing congenital deafblindness. Therefore, the research gave a snapshot of the current knowledge of these professionals in the management of the deafblind population. This is the first study that has investigated the competence and confidence of the rehabilitation healthcare professionals in differentially diagnosing deafblindness from ASD.

## Conclusion

The misdiagnosis or late diagnosis of deafblindness has lifelong consequences for the family and the child who is deafblind. Therefore, there is a need to prioritize early identification and intervention in this population. The study highlights the implications at three different levels:

### Rehabilitation practice

There is a need for continuous education of the rehabilitation healthcare professionals and peer learning to equip them with the information and skills they need to successfully identify and manage children who are deafblind. This would potentially improve the quality of early identification processes and facilitate a more detailed and accurate diagnosis of the condition. Multi-disciplinary training opportunities for healthcare professionals are highly recommended. Even though the prevalence of deafblindness is low compared to other multisensory disorders, a deliberate effort is needed for healthcare professionals working with this population to create awareness regarding the condition of deafblindness.

### Policy

In low-and-middle-income countries, specifically in South Africa, deafblindness is not identified as a separate condition. Therefore, the findings of this study call for recognition of deafblindness as a separate disorder. It should not be grouped with other neurodevelopmental disorders which often closely resemble deafblindness. This will also assist in identifying resources and reasonable accommodation needed for this population.

### Research

There is a need for further research that could facilitate the development of deafblindness specific services that would benefit both the child who is deafblind and their families. Research in the field of deafblindness would also ensure that intervention strategies implemented for children who are deafblind are evidence based.

## Data Availability

All data generated or analyzed during this study are included in this published article.

## Abbreviations

ASD: Autism Spectrum Disorder
CPD: Continuous Professional Development
HPCSA: Health Professions Council of South Africa
HREC: Human Research Ethics Committee
OTASA: Occupational Therapy Association of South Africa
PASA: Physiotherapy Association of South Africa
SAAA: South African Audiology Association
SASLHA: South African Speech Language and Hearing Association
WMA: World Medical Association
